# The plasmid-host fitness landscape: a new paradigm for predicting the fate of mobile resistance

**DOI:** 10.1128/aem.01983-25

**Published:** 2026-01-05

**Authors:** Hevar N. Abdulqadir

**Affiliations:** 1 MLS Department, Komar University of Science and Technology 449486, Sulaymaniyah, Iraq; The Pennsylvania State University, University Park, Pennsylvania, USA

**Keywords:** plasmid-host fitness landscape, antimicrobial resistance (AMR), plasmid fitness cost, compensatory evolution, horizontal gene transfer (HGT), co-evolution

## Abstract

The widespread persistence of antimicrobial resistance (AMR) plasmids presents a fundamental challenge to microbial evolution, known as the “plasmid paradox”: if these plasmids cause fitness cost, why are they not eliminated by selection? The classical view, which imposed a fixed generic fitness cost, is insufficient to explain their epidemiological success. Here, we propose a new paradigm—the plasmid-host fitness landscape—a multi-dimensional model that takes into account the complex interplay between ecology and genetics. This landscape unfolds into three main axes. First, the host axis reveals that fitness costs often arise from host-dependent genetic conflicts, not a generic burden. Second, the time axis demonstrates that the fitness cost of any plasmid can be negated over time through plasmid or chromosome compensations, which leads to ameliorating initial costs and locking in resistance. Third, the environmental axis shows that the fitness cost of any plasmid can be affected by external factors like temperature and sub-inhibitory concentrations of antibiotics. These factors dynamically modulate the benefits and costs of plasmid carriage. By integrating the complex interplay between these dimensions, we argue that the plasmid fitness costs are not a fixed generic measurement, but rather a contingent trajectory across this landscape. This paradigm shifts the focus from static measurements to a dynamic, predictive science, providing a new foundation for assessing and managing the threat of mobile resistance.

## INTRODUCTION

Plasmids and other mobile genetic elements (MGEs) are essential for bacterial evolution and adaptation to environmental stress. They also act as the primary vectors for horizontal gene transfer (HGT) (1–3). Their most profound clinical impact is the transmission of antibiotic resistance genes (ARGs), which allows pathogens to develop resistance to life-saving antibiotics, exacerbating the global health crisis (4, 5). Regardless of how essential these genetic factors are in the presence of antibiotics, their carriage is not without consequence (6). In the absence of direct selection, plasmids have been shown to have a deleterious effect on their host. Under safe environmental conditions, plasmid-carrying bacteria typically exhibit reduced growth rate compared to their plasmid-free counterparts (6, 7).

This negative impact of plasmids raises the long-standing question in microbial evolution known as the plasmid paradox: if these genetic elements are inherently costly and have negative side effects, why are they so ubiquitously maintained in diverse bacterial populations, even in environments where selection for their cargo genes is intermittent or absent? (6, 8). The initial fitness cost of plasmid acquisition creates a barrier to evolution, limiting both vertical inheritance and horizontal transmission (6). Therefore, understanding the mechanisms that allow plasmids to overcome this initial burden is critical for comprehending and predicting their long-term persistence and epidemiological success (9, 10).

Early hypotheses proposed that this fitness cost arose from generic burden, such as the energetic demands needed for DNA replication, or translational load that came from these plasmids with non-native codon usage patterns (6, 11). However, this view was proven to be insufficient. A systematic investigation of diverse clinical plasmids in *Escherichia coli* demonstrated that plasmid burden is not necessarily linked to stress caused by translational machinery, as a mutant host with reduced ribosomal activity showed no translational competition and also no increase in plasmid-associated burden (11). This provided strong evidence against the original theory. This finding moved the field beyond simplistic models of a generalized metabolic drain, compelling a re-evaluation of the specific molecular interactions that truly underpin the plasmid-host relationship.

**Fig 1:**
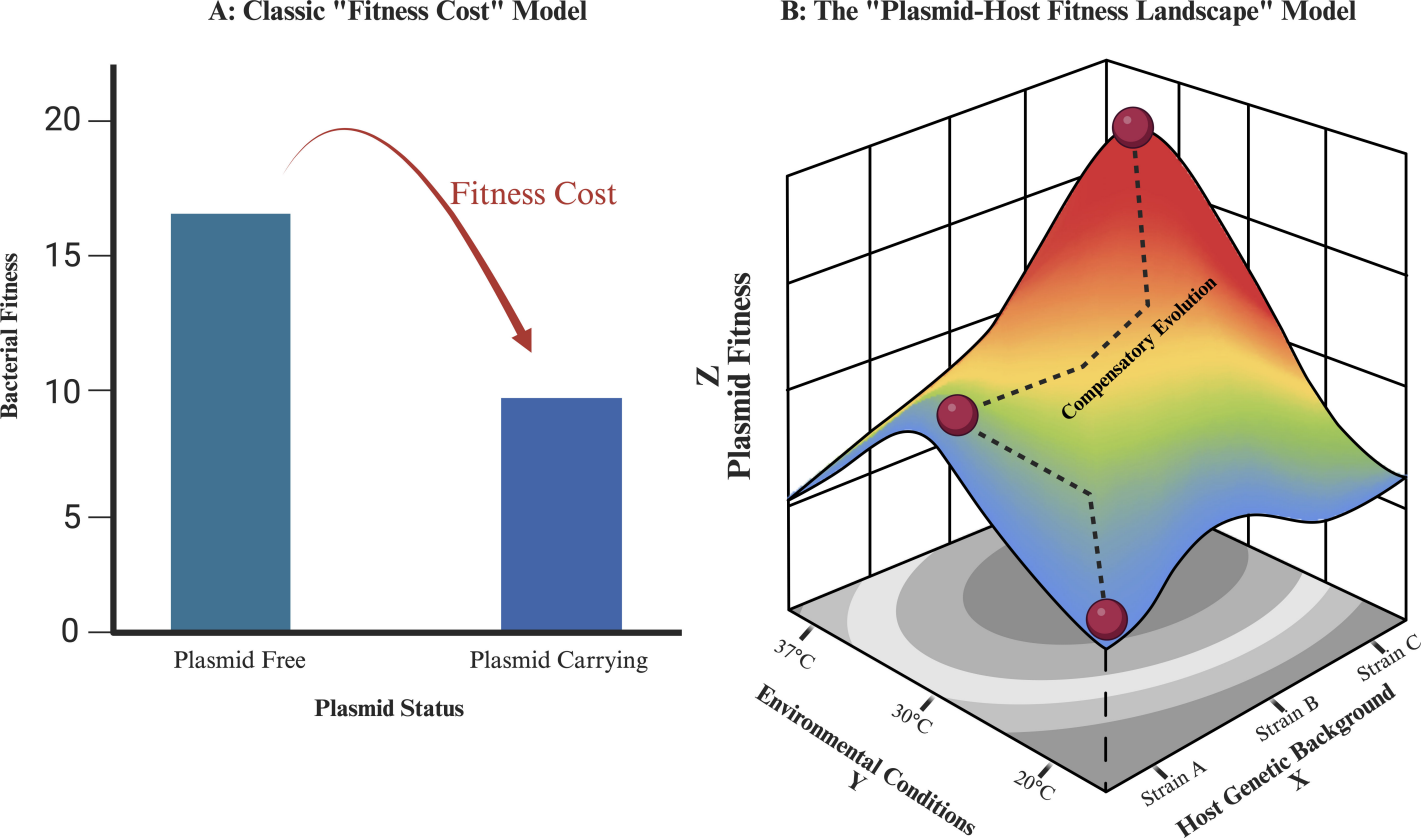
The plasmid-host fitness landscape model. (**A**) Classic fitness model. Plasmid carriage is viewed as imposing a fixed, deleterious effect, which reduces the competitive fitness of the host bacterium relative to its plasmid-free counterpart. (**B**) Plasmid-host fitness landscape model, which shows that plasmid fitness depends on a complex interplay between environmental conditions (Y-axis), host genetic background (X-axis). Any given plasmid (red dots) can be high cost, neutral, or beneficial depending on its location in this landscape. The dashed line between the red dots shows compensatory evolution, which allows a host to move from a low fitness “valley” to a more neutral region through mutation. Created in BioRender.

Resolving this plasmid paradox requires a more sophisticated, multi-dimensional framework. Here we propose a new paradigm named the—plasmid-host fitness landscape—which is a conceptual framework that accounts for the complex and intricate interaction between the plasmid and its host genetic background, their co-evolutionary trajectory, and also their physicochemical environment (Fig. 1). By synthesizing recent advances, we will argue that the fate of mobile resistance genes is not determined by a single factor but rather by its contingent position and trajectory across this multi-dimensional landscape. This review will deconstruct the key axes of this landscape to provide a better understanding of this topic and to ultimately help in predicting the future of mobile resistance.

## THE FIRST DIMENSION: THE HOST AXIS

The cost of a newly acquired plasmid is not a fixed property of the element itself. Rather, it is a complex relationship between the plasmid and the genetic makeup of its host (Box 1) (12). This understanding leads to a new paradigm that shifts away from the early models that associated fitness cost with generic and non-specific burdens (13). The new model, supported by a wealth of recent evidence, suggests that the fate of any plasmid depends on the specific genetic context of the host it inhabits (10, 14–16).

Box 1. **How is plasmid fitness cost experimentally measured?**

The fitness cost of a plasmid is the measure of the burden that the plasmid is having on its host (16). The fitness cost is measured using quantified methods like head-to-head competition assay (16). The strain carrying the plasmid is put into a competition against its genetically identical plasmid-free counterpart. This experiment allows for the measurement of the net effect of the plasmid on the host reproductive success (17).

The standard experiment involves mixing the two strains in a 1:1 ratio in liquid culture. Then the mixed population is propagated for a set number of generations through daily dilution and serial transfer. Samples from this liquid culture are taken at the start of the experiment and at the end, then the ratio of plasmid-free cells to plasmid-carrying cells is measured (17).

Modern assays have revolutionized the precision of this technique. Competing strains are often engineered to express different fluorescent proteins (e.g., GFP and mCherry), allowing for rapid and accurate counting of millions of individual cells using flow cytometry (18). The relative fitness of the plasmid-carrying strain is then calculated from the change in its frequency relative to the plasmid-free strain over the course of the experiment. This high-throughput method allows for the detection of extremely subtle fitness differences, often less than 1%, which are critical for understanding the long-term evolutionary fate of plasmids (18).

### From generic burden to specific genetic conflicts

The inability of the early models to fully account for observed fitness cost had led to their obsolescence (13). The claims of these old models and their central prediction that the cost of the plasmid should scale as its size becomes larger or the number of genes it encodes have been frequently contradicted by experimental data (3, 14). For instance, recent experiments have shown that analysis of a large plasmid that carries multi-resistance genes revealed no clear correlation between the plasmid size or how many resistance genes it carries and the fitness cost imposed on the host (13). Similarly, another study demonstrated that a clinically significant plasmid, *IncX3,* which carries the *bla*NDM, can impose significantly different fitness costs on various host strains, a variability that cannot be explained by the generic models (17, 18).

Perhaps the most convincing evidence against the generic models is the compensatory evolution. If the fitness cost were the result of multifactorial load, then its amelioration would require a significant amount of evolutionary adjustments (17). Yet many recurring studies have demonstrated that the fitness cost of even a large plasmid can often be completely negated with one targeted compensatory mutation (19). This rapid resolution strongly points to a singular, specific point of conflict rather than a generalized metabolic load (20).

These discoveries have led to the establishment of the “specific genetic conflict” model. This model suggests that fitness costs arise from specific negative interactions between the discrete plasmid and the chromosomal genes (6, 17, 19). Mechanistic proof for this model is now robust. A landmark study conducted on *Pseudomonas fluorescens* demonstrated that the presence of two mega-plasmids caused a significant fitness cost that was caused by a single conflict: the inappropriate induction of the chromosomal tailocin toxin operon (17, 19). Compensatory mutations, like reducing the expression of the host gene *PFLU4242,* were sufficient to completely eliminate the cost (17, 19).

While the specific genetic conflict model explains the nature of plasmid fitness cost, it is also noteworthy to mention that the magnitude of this burden is greatly affected by the plasmid copy number (21, 22). The average number of plasmids per cell is a tightly controlled trait that determines the overall dosage of the plasmid-encoded genes (21, 23). A higher copy number of the plasmid can be a double-edged sword for its host. Under selective pressure, it can increase the resistance of the cell, or it can increase metabolic load on its host by increasing resources needed for DNA replication and gene expression (11, 21). This creates a fundamental trade-off where the benefit of gene dosage is balanced against the cost of the burden (23). Crucially, plasmid copy number is not a fixed generic burden but rather a dynamic trait that is affected both by the external environment and the host genetic makeup (23, 24). Therefore, to determine the initial fitness cost of a plasmid, one needs to not only account for the specific genetic conflict but also account for the quantitative impact of the plasmid copy number within that specific host and environment.

### Host specificity: the same plasmid, different fates

A profound and direct consequence of the specific genetic conflict model is that the fitness cost of any given plasmid is highly host dependent. The fitness cost of any plasmid happens because of the gene-for-gene interactions. It logically follows that the outcome of any interaction depends on the alleles that are present in the host genome. This explains the phenomenon where one plasmid is costly for one strain, neutral in another, and even beneficial in a different strain (10, 12).

**Fig 2:**
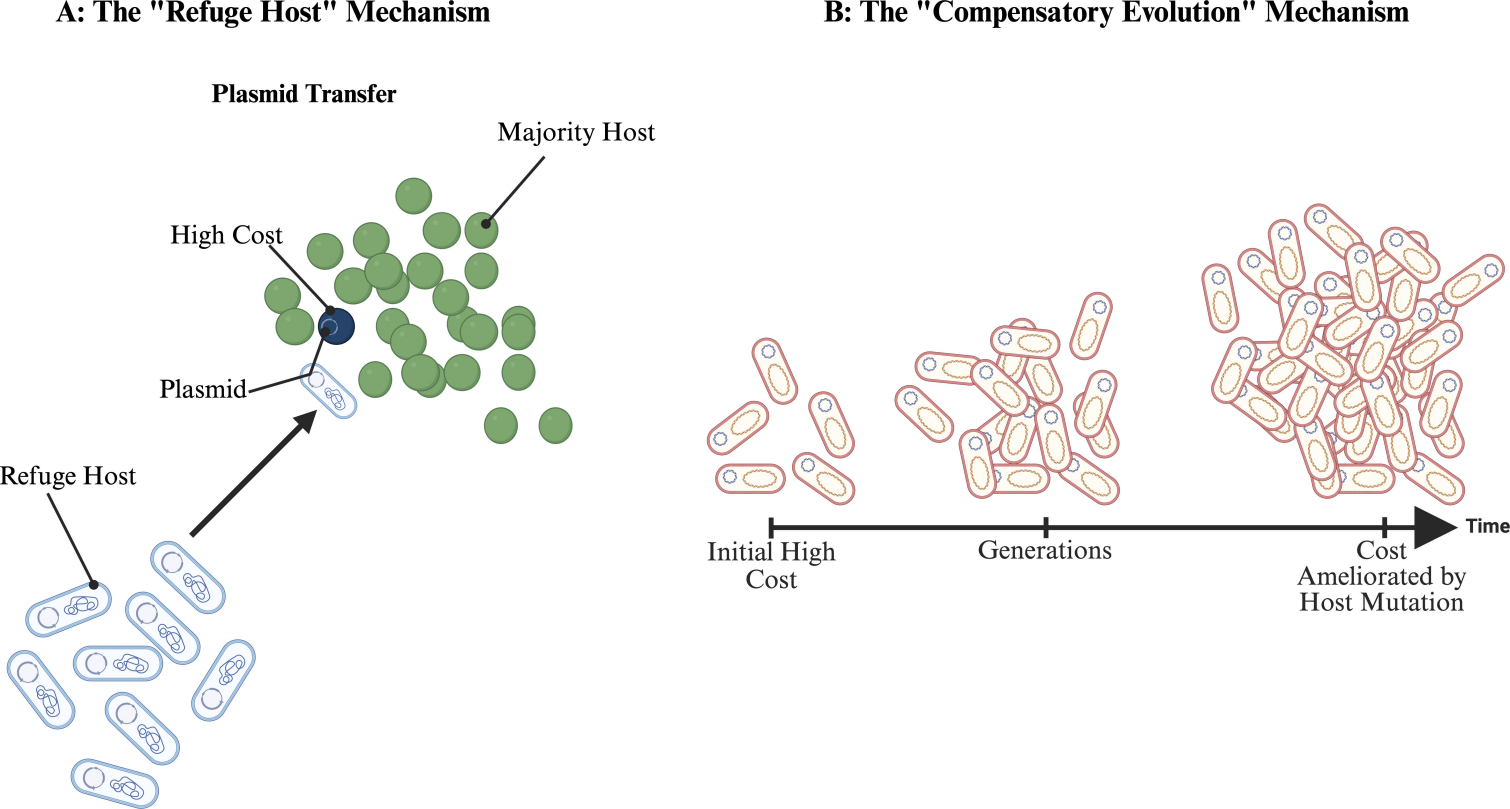
Key ecological and evolutionary dynamics. The figure shows the ecological and evolutionary dynamics that shape this landscape. (**A**) The left side of the panel showcases the refuge host mechanism, where a minority of the host population (blue oval) can maintain the plasmid at low cost, acting as a stable supply source for the majority of the population (green oval), where the same plasmid is costly. (**B**) The right side of the panel shows the process of compensatory evolution, which states that a host that acquires a costly plasmid is initially unfit, but over successive generations and through mutation that ameliorates this cost, stabilizes the plasmid in the population. Created with BioRender.

All the data that have been compiled from recent studies (as summarized in the literature synthesis [Table 1]) provide strong validation for this principle. For instance, a systematic study demonstrated that six cloned ARG genes that were placed in a common vector showed very different fitness costs across eleven diverse *E. coli* strains; each of these ARG genes was costly in one strain and beneficial or neutral in others, demonstrating the existence of selective refuge (Fig. 2) (12). This variability was also seen in clinically relevant plasmids. An *IncX3* plasmid, which carries the *bla*NDM genes, was proven to be costly for the *E. coli* C600 yet showed no noticeable fitness cost on *E. coli* J53, which are both common lab strains (17). Therefore, this context dependency is a crucial factor in predicting the persistence of any plasmid (10).

**TABLE 1:** Key mechanisms of compensatory evolution for plasmid fitness cost amelioration

Locus of mutation	Characteristics of compensatory mutations				
	Gene/region	Mechanism of action	Model system	Reported trade-off(s)	Reference
Chromosome	*gacA*/*gacS*	Alters global host transcription	*P. fluorescens* + *pQBR103*	Potential loss of other Gac-regulated functions	(25)
Chromosome	*PFLU4242*	Prevents the maladaptive expression of a host toxin operon	*P. fluorescens* + *pQBR57*	None reported	(21)
Chromosome	Metabolic and membrane genes	Alters host metabolism and membrane function	*E. coli* + *IncX3* plasmid	Not fully characterized	(19)
Plasmid	*upf31*	Inactivates a costly plasmid regulatory gene	*E. coli* + *pBP136* Km	None reported	(20)
Plasmid	IS-mediated deletion	Deletes costly modules (e.g., AMR genes)	*E. coli* + MDR plasmid	Reduced/lost conjugation ability; loss of resistance	(9)

### Co-evolution and pre-adaptation in clinical settings

The fact that fitness cost depends on the host has a critical implication in clinical settings. The global spread of some of the most threatening resistance genes is often driven by the expansion and spread of highly specific successful pathogen clones that appear to have pre-adapted or co-evolved to carry the plasmid with minimal cost (10, 26).

A clear example of this phenomenon is the relationship between the globally distributed *blaKPC* resistance gene and its typical host, *Klebsiella pneumoniae* sequence type 258 (ST258) (26). When the plasmid carrying *blaKPC*(*pKpQIL*) was studied in *K. pneumoniae*, it was found that the plasmid was stable and showed no noticeable burden (26). This state was not achieved by countless genetic mutations but rather by rapid transcriptional adaptation. Upon acquiring the plasmid, the host cell undergoes a rapid reprogramming of its entire gene expression profile, affecting both the plasmid and chromosomal genes to achieve a state of physiological harmony (27).

This “software” fix represents a sophisticated adaptation system that is both fast and invisible to standard genomic sequencing techniques (27). It fundamentally challenges the dogma that a plasmid will inevitably be lost once antibiotic selection is removed (10, 27). This co-evolution between the plasmid and the host allows them to be persistent in the default state, allowing them to act as a reservoir for the spread of antibiotic resistance genes (10, 27). This highlights a major deficiency in surveillance programs that only account for the plasmid sequence to predict risk. It is also necessary to account for the co-evolved plasmid-host pair (10).

## THE SECOND DIMENSION: THE TIME AXIS

**Fig 3:**
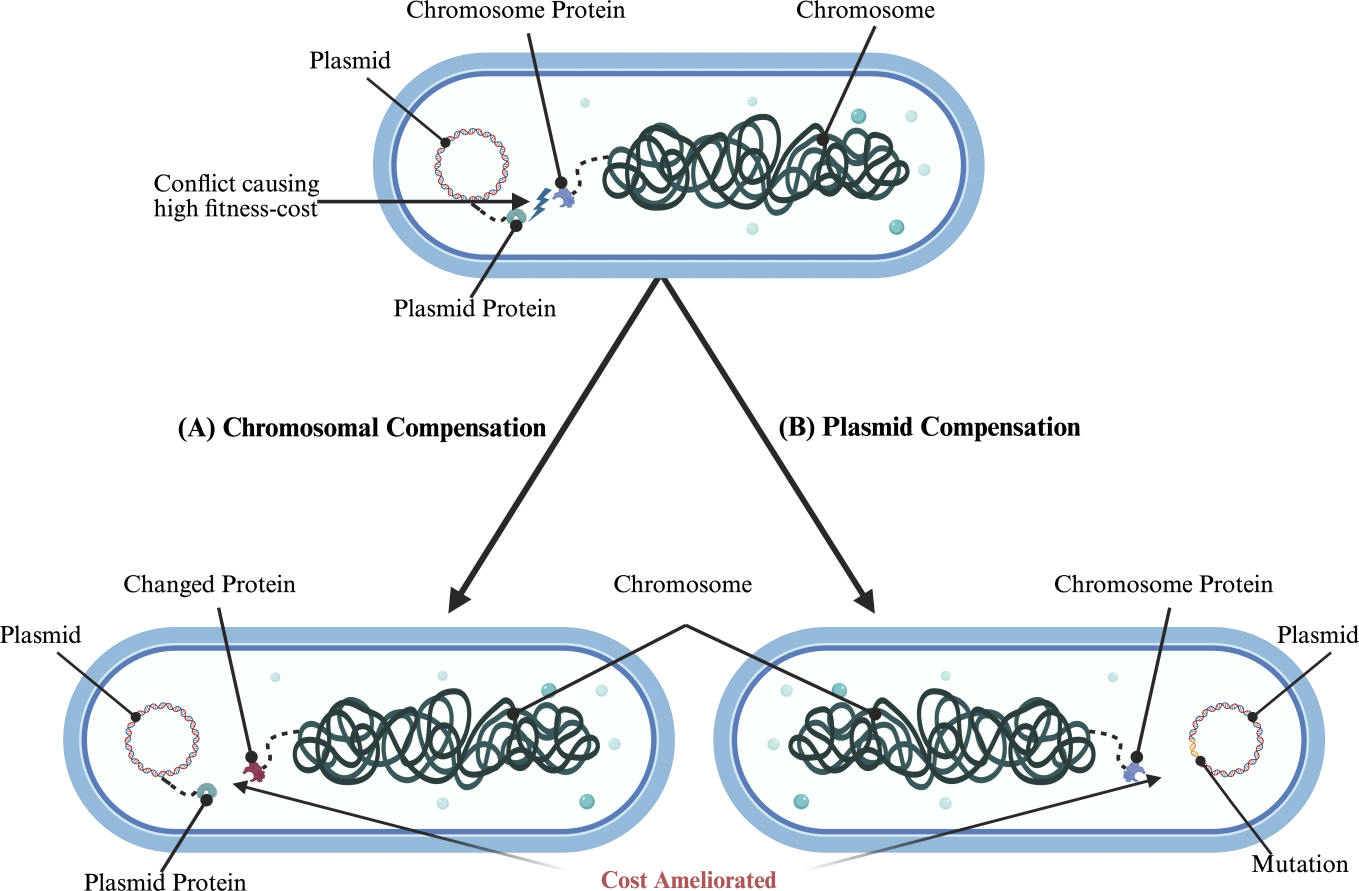
The molecular mechanisms of compensatory adaptation. A schematic illustration showcasing the two pathways to reduce the fitness cost of a plasmid. (**A**) In chromosomal compensation (chrCMs), a mutation (indicated by a star) happens on the host chromosome that resolves the genetic conflict between the host and the plasmid. (**B**) In plasmid-level mutation, the changes happen at the plasmid level (plaCMs), resulting in a reduction in fitness cost. Both pathways ultimately lead to reduced fitness cost and the persistence of the plasmid in the population. Created with BioRender.

The solution to the plasmid paradox unfolds along the time axis. The initial fitness cost imposed by the plasmid on its host is not permanent; rather, it is the start of a dynamic co-evolutionary race between the host and the acquired plasmid (22, 28). Over generations, the process of compensatory evolution mitigates the initial burden of the plasmid, paving the way to a stable long-term association (22, 29). This adaptive process happens alongside two completely different pathways that sometimes even compete, defined by their location or locus of the compensatory mutations (CMs) (30). Either the plasmid can adapt to become less burdensome, resulting in plasmid compensatory mutations (plaCMs), or the host can adapt by altering its chromosome, leading to chromosomal compensatory mutations (chrCMs) (Fig. 3) (30). Understanding how these pathways unfold is key to determining how a costly plasmid gets locked into a bacterial population (22).

### The host strikes back: chromosomal compensatory evolution

One of the most common adaptive responses to the fitness cost of a plasmid is for the host to mutate its chromosome, so that it accommodates the extra genetic element through recalibration of the cellular pathways. A common strategy includes mutations in global transcriptional regulators. A change in this master switch allows the host to alter the cellular physiology to better fit the plasmid (29). An excellent example of this is *GacA/GacS*, a two-component system in *P. fluorescens,* where mutation in either *GacA*~*GacS* results in complete resolution of any burden caused by conjugative mega-plasmid, a phenomenon observed both in wet lab experiments and in complex natural environments, such as the plant rhizosphere (29).

Alternatively, chromosomal compensation can also happen through highly specific targeted mutations that aim to resolve the conflict between the plasmid and the host, leading to reduced cost. As it was stated in the specific genetic conflict model, often the majority of the cost caused by any plasmid is the result of a single negative interaction between the chromosome and the plasmid (19). For example, the fitness cost of the mega-plasmid in *P. fluorescens* was found to be caused by a single conflict between the chromosome tailocin operon and the plasmid, a conflict that could be resolved completely by a single targeted mutation in the host gene *PFLU4242* (19). In a similar study, it was demonstrated that the cost of the *bla*NDM-carrying plasmid in *E. coli* stemmed from the conflict with host metabolic and membrane function genes, and these costs were compensated by mutations in the pathways controlling oxidative stress and metabolism (17).

### The plasmid adapts: intrinsic mechanisms of amelioration

The plasmid itself is also a potent target for mutation and evolution. The mutations that happen at the plasmid level (plaCMs) are particularly powerful (29). They solve the fitness cost issue in the host cell and benefit the survival of the plasmid. These mutations result in a plasmid that is no longer a burden on any new host during conjugation (29, 31).

One of the most direct strategies that plasmids use to reduce cost is evolution by subtraction. This process involves the plasmid shedding the portion that is costly for the host (9, 29). These targeted deletions often remove genes that are metabolically expensive, like conjugation machinery or accessory genes like antimicrobial resistance cassettes that are not under selection (7, 9). These events are often controlled by insertion sequences (IS elements), which are hot spots for genetic recombination or rearrangement in response to selective pressure (7, 9).

A more precise strategy is the inactivation of a single host-antagonistic gene on the plasmid (29). The absolute example of this strategy is the gene *upf31* on the *IncP-1β*p*pBP136* (20). It was shown that this single gene was completely responsible for a high fitness cost in *E. coli*. Through the deletion of this single gene, the high fitness cost was completely resolved and resulted in a stable plasmid (20). This new mechanism that determines the activity of plasmid genes provides a clear pathway for host-range expansion, as a single mutation could unlock a new species for long-term persistence (32).

### Intrinsic stability mechanisms: toxin-antitoxin systems

**Fig 4:**
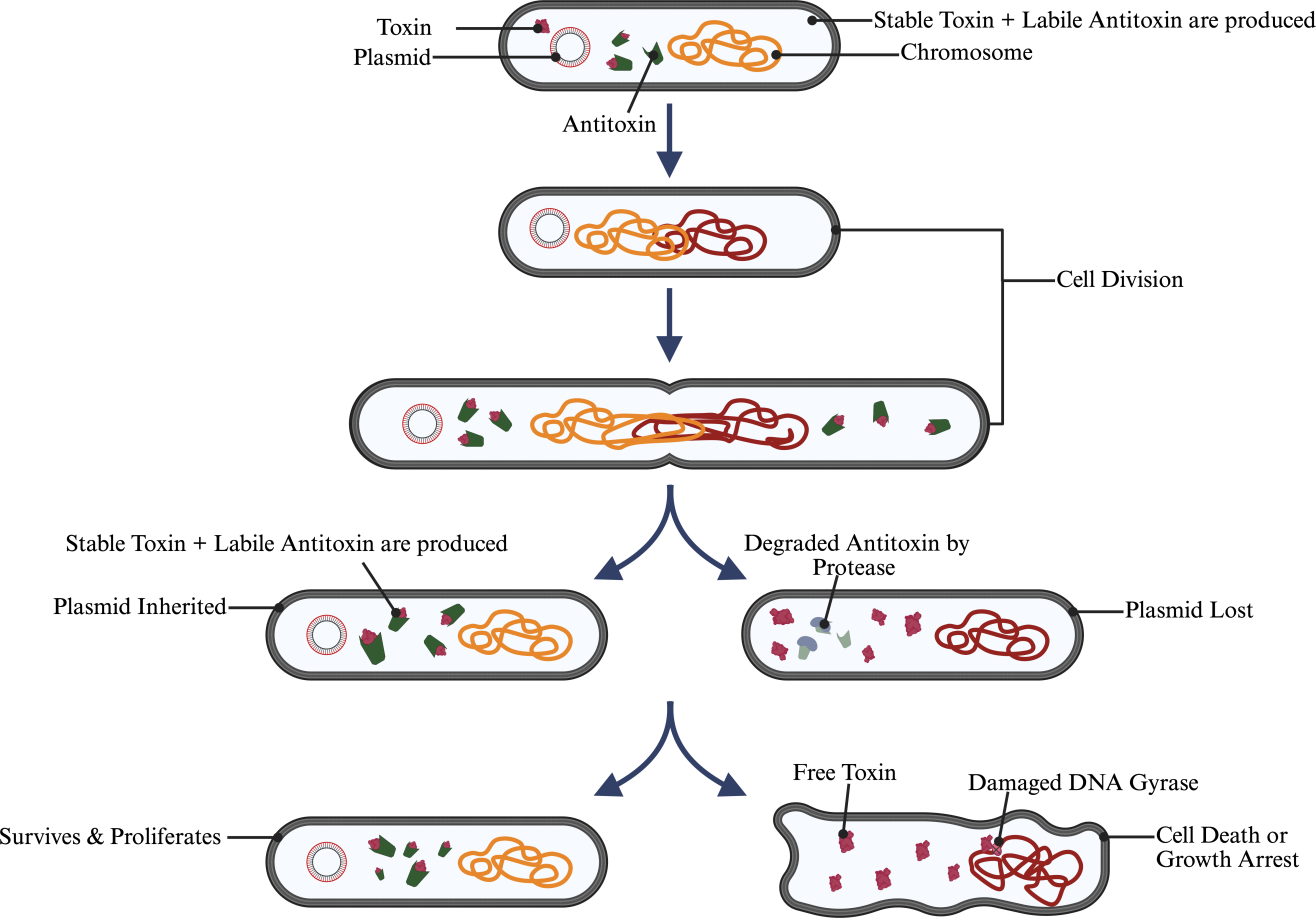
The toxin-antitoxin (TA) "addiction module" mechanism for plasmid maintenance. An illustration that shows the type II toxin-antitoxin system that ensures plasmid survival through PSK. The top shows the parent cell that possesses the plasmid, creating both a stable toxin and a labile antitoxin protein. (Bottom left) After cell division, the daughter cell that inherits the plasmid goes on producing both components and survives. (Bottom right) When a daughter cell fails to inherit the plasmid, the cell no longer produces a labile antitoxin. The unstable antitoxin rapidly gets degraded by the host cell protease, leading to the accumulation of free toxin. Eventually, the toxin will attack essential cellular components like DNA gyrase, resulting in growth arrest or cell death. This process selectively eliminates plasmid-free cells from the population. Created in BioRender.

Beyond the ways the host and plasmid adapt to ameliorate fitness cost, many plasmids ensure their survival through a more direct and coercive strategy: the development of toxin-antitoxin (TA) systems. These systems are often referred to as “addiction modules,” which result in stable inheritance of the plasmid throughout generations by a mechanism known as post-segregational killing (PSK) (Fig. 4) (33–35). The presence of these systems is like having an evolutionary insurance policy. While they do not prevent the physical loss of the plasmid during cell division, they instead kill any daughter cells that fail to inherit it. Through this process, the plasmid ensures to eliminate any potential competitors and the successful survival of the plasmid lineage (34, 36). TA systems are especially important for low-copy-number plasmids, which are at higher risk for segregational loss (34). While TA systems were first discovered on plasmids, they are now understood as selfish, ubiquitous genetic modules that are present on many bacterial chromosomes and other MGEs (37–39). They function as a ruthless quality control system that prevents and punishes plasmid loss and destroys the evidence of segregational loss, a process that is triggered by the failure of the primary active partitioning machinery (e.g., *ParABS* systems) (35).

The efficacy of any PSK systems greatly depends on the differential stability of two of the components of the system: the toxin and the antitoxin (35, 36). In cells that harbor the plasmid, the antitoxin, often made of protein or RNA, continuously neutralizes the toxin, ensuring the safety of the cell. On the other hand, plasmid-free daughter cells have unstable antitoxin due to continuous degradation by the host proteases, which in turn results in leaving the toxin free to attack essential cellular components, leading to growth arrest or cell death (33, 40). The varieties of these systems are vast and based on the nature and the action of the antitoxin component, at least eight varieties have been identified (38, 41). In the type I TA system, the antitoxin is an RNA antisense which inhibits the translation of the toxin’s mRNA (34, 36). Type II TA systems, which are more commonly seen, have a protein antitoxin that binds to and sequesters the toxin protein (38, 42, 43). Other types utilize more exotic mechanisms, such as an RNA antitoxin binding a protein toxin (type III) or an antitoxin that protects the toxin’s cellular target (type IV) (34, 44).

Recent studies on the impact of TA systems on the persistence and spread of antimicrobial resistance (AMR) show that they are clinically relevant (45). For instance, a 2024 study conducted on *Shigella sonnei* highlights that if a single mutation happens in the *VapB* antitoxin of the *VapBC* TA system, it will lead to a more stable *pINV* virulence plasmid. This mutation not only increases the lability of the antitoxin component but also accelerates the degradation of the antitoxin, leading to a state of dormancy that, under antibiotic stress, confers antibiotic tolerance to the host (45). This creates a powerful synergy where selection for either plasmid stability or antibiotic tolerance enriches for the same mutation. Another study conducted on the pConj plasmid of *Neisseria gonorrhoeae* found that while the plasmid carries an “orphan” VapD toxin, it lacks a cognate antitoxin on the same replicon. This toxin is neutralized with VapX antitoxin, which exists as a separate genetic element in the same cell. This split TA system creates a strong selective pressure where cells with both genetic elements survive, which helps to explain the narrow host range of some AMR plasmids (46). These mechanistic studies, alongside genomic analysis, all converge on the conclusion that the presence of TA systems is tied to the persistence of AMR genes. These findings confirm that TA systems are a critical component of the time axis, acting in parallel with compensatory evolution to ensure the long-term persistence of mobile resistance (39).

### Restriction-modification systems as analogous maintenance modules

Another mechanism that the plasmids use, which is a functionally analogous “addiction” system, is the use of the restriction-modification (R-M) system (47). This modification system was initially known as an innate bacterial immune response against bacteriophage (48); foundational studies in 1995 found that plasmids with R-M systems can also act as highly effective plasmid maintenance modules (49, 50). Just like the TA system, R-M plasmids ensure their survival through PSK (50, 51).

The R-M system is a clear example of convergent evolution, repurposing a host defense system into a “selfish” system (49). This model is an analogous example of a type II TA system, where the toxin is the stable restriction endonuclease (REase) and the antitoxin is the labile modification methyltransferase (MTase) (50, 51). A cell that carries the plasmid continues to make MTase, which in turn adds protective methyl groups to all the self-recognition sites on the chromosome, marking these areas as self and protecting them from the action of the REase (50, 51). However, if a daughter cell fails to inherit the plasmid with the R-M system, the cell stops making labile MTase. When the existing MTase gets degraded, the stable REase persists (51, 52). The stable REase then recognizes the unmethylated regions in the chromosome, introducing lethal double-strand breaks in the host chromosome, leading to cell death (50, 51).

These similarities between the TA and R-M systems as selfish genes are now well established in the literature (47, 49, 53). While it is true that their molecular evolution is unrelated, their way of surviving has converged on the same mechanism of having a stable toxin and labile antitoxin to enforce their own inheritance (47). Modern research even contextualized both systems under the broader umbrella of “MGE selfishness,” where the genetic mobile element acts to safeguard its own survival and persistence, leading to the exclusion of competing MGEs (54, 55). The discoveries of both TA and R-M systems provide a more complete picture of the diverse and sophisticated strategies that plasmids employ to ensure their survival over evolutionary time.

### The dynamics of co-evolution: locus, trade-offs, and ecology

The existence of these two distinct pathways raises a central question: which strategy is evolutionarily superior? Based on theoretical models, plasmid compensatory mutations (plaCMs) have been seen as superior, due to their advantages during HGT (30, 31). However, experimental studies have repeatedly shown that chromosomal compensatory mutations (chrCMs) are often more dominant than plasmid-level mutations (30). The paradox between these models can be resolved by considering the magnitude of the fitness benefit; a highly effective chromosomal mutation can provide an extreme growth advantage that allows it to outcompete the slow-growing plaCM lineage, even if the plaCM lineage has a theoretical long-term transmission advantage (30).

This co-evolutionary process is further driven by evolutionary trade-offs. The most critical of these is the conflict between horizontal transmission (plasmid survival) and vertical transmission (host growth) (22). The expression of costly conjugation machinery can slow down the growth of the host, which is a trade-off that a plasmid must navigate (56, 57). Based on lab experiments, it has been shown that many plasmids prioritize vertical transmission, while others even go further and sacrifice infectivity by deleting the entire conjugation machinery genes to maximize host fitness (9, 56, 57).

Finally, these adaptation strategies have profound ecological consequences (58). Hosts that mitigate plasmid fitness cost through mutation can deploy the plasmid effectively as a biological weapon. Transferring this still-costly plasmid to other hosts and burdening them with fitness cost is a strategy known as plasmid weaponization (30). These techniques that some bacterial strains use pose a significant challenge. The compensated strains can act as stable hubs for antibiotic resistance genes throughout a microbial community (30).

The diverse mechanisms of both chromosomal and plasmid-level compensation discussed in this section are summarized in Table 1.

## THE THIRD DIMENSION: THE ENVIRONMENTAL AXIS

The plasmid fitness landscape is not a fixed map that only relies on genetics and time alone. It is a dynamic landscape that is influenced by a third critical factor: the external environment. A plasmid-host pair that is costly in one condition can be extremely efficient in another. This context dependency is a complex interplay between physical, chemical, and nutritional factors, which are essential in determining the fate of mobile resistance (59–61).

### Chemical stressors: the paradoxical role of sub-inhibitory antibiotics

Far from being inert, sub-concentrations of antibiotics that are too low to kill bacteria are prevalent in clinical and natural environments (60, 62). This alone acts as a potent signaling molecule for the spread of antibiotic resistance genes. A substantial body of evidence has shown that exposure to a wide array of antibiotics like β-lactams, fluoroquinolones, and polymyxins can significantly increase the frequency of plasmid-mediated conjugation (62, 63). For instance, the last-resort antibiotic colistin has been shown to increase the frequency of plasmid-mediated conjugation for plasmid-carrying *mcr-1 *and *bla*NDM-5 genes up to 10- and 8-fold, respectively (64). Also, exposure to sub-MIC concentrations of ciprofloxacin has been shown to increase plasmid transfer rates by almost ninefold (63). This phenomenon creates a dangerous positive feedback loop where the presence of the antibiotic increases the transfer rate of the very plasmid that confers resistance to it.

This sub-MIC concentration not only increases transfer rates; it can also completely alter the fitness landscape. It has been demonstrated that the cost of carrying any plasmid can be negated in the presence of low levels of antibiotics (60). Competition experiments have demonstrated that plasmid-carrying cells grow much faster in environments that have as low as 1/20 of the MIC concentration when compared to their plasmid-free counterparts (65). This provides strong evidence for the plasmid maintenance and persistence in the population (65).

### The SOS response: a mechanistic hub for stress-induced evolution

The molecular mechanism that often links antibiotic stress to HGT is the SOS response system, a conserved global regulatory system that gets activated in response to DNA damage (66–68). Many antibiotics, especially those from the fluoroquinolone class, have been known to induce DNA damage and are thus potent activators of the SOS pathway (63, 69). This activation is critical because of the direct regulatory effect on integrons, which are genetic platforms that capture and express ARG cassettes (70).

The promoter for the integron integrase gene (*intI1*) often contains a binding site for the SOS repressor protein, *LexA* (69, 71). When antibiotic stress activates the SOS response, the *LexA* repressor is cleaved, leading to high-level expression of integrase (69, 72). This sudden high-level expression of integrase allows bacteria to sample different gene configurations to find the optimal condition for the expression of the ARG genes, a phenomenon known as evolution on demand (73). This mechanistic link has been shown to be clinically relevant, as studies have shown that antibiotic therapies could lead to SOS response and activation of integrase, which could lead to the evolution of new resistance arrays during treatment (74).

### The influence of physical and nutritional landscapes

The plasmid fitness landscape is affected beyond chemical stressors; nutritional and physical environments of the host have been shown to greatly affect this complex interplay (59–61). Temperature is a key modulator. It has been shown that many clinically relevant plasmids are thermoregulated, exhibiting optimal transfer rates not at human body temperature (37°C), but rather at cooler ambient temperatures (25°C–30°C) (75). This behavior showcases that the transfer of clinically relevant plasmids (like carrying *blaKPC *and *bla*NDM) is actually not happening in the infected patients; rather, it happens in external reservoirs like hospital wastewater systems (75). This mechanism of transfer provides an explanation for the observed seasonality of some Gram-negative infections, as conditions in the environment become optimal for plasmid transfer during the hot summer months (59, 76).

The bacterial lifestyle—specifically in planktonic state vs growth within biofilm communities—also creates a drastically different environmental condition for HGT. The close proximity and cell density seen in biofilm communities create hot spots for conjugation, with transfer rates being the orders of magnitude higher than in planktonic cells (77). Furthermore, the reduced growth rate of some bacterial cells deep within the biofilm communities creates the optimal conditions for plasmid persistence by preventing segregational loss, making biofilm communities a stable reservoir for resistance genes (78–80).

Finally, the entire process is limited by nutrient availability. The process of conjugation is extremely energy-intensive (61), and numerous studies have shown that in low nutrient conditions, plasmid transfer rates are significantly reduced compared to those in nutrient-rich laboratory conditions (81). This highlights a critical caveat for many lab-based studies, as transfer rates measured under optimal conditions may grossly overestimate the potential for plasmid dissemination in natural, oligotrophic environments (81).

## SYNTHESIS: THE PLASMID-HOST FITNESS LANDSCAPE AS A PREDICTIVE FRAMEWORK

The research synthesized in this review all converges on a new multi-dimensional paradigm, one that makes it possible for understanding the fate of mobile resistance. The point of view of older models, which proposed a single fixed fitness cost, is insufficient for predicting the fate of antibiotic resistance genes in the wild (6, 13). We deconstructed this complex problem along three axes—Host, Time, and Environment. Here, we formally define the plasmid-host fitness landscape model, which accounts for all these axes, and propose a new predictive approach for predicting the fate of mobile resistance in nature.

### Defining the new paradigm

The plasmid-fitness landscape is a dynamic model that explains the fitness cost of any plasmid, and it demonstrates that the burden of a plasmid is not a single fixed, predetermined value but a contingent state. The key axes of this landscape include the following.

1. The host axis: the genetic background of the host is one of the foundational dimensions. As it was showcased with previous examples, often plasmid fitness cost is caused by the antagonistic interactions between the host genetic and the plasmid. Thus, a plasmid fitness cost is, consequently, host-dependent, ranging from harmful to neutral and in some strains even beneficial (10, 12, 22).
2. The time axis: time is another foundational dimension that makes this landscape not static, but rather it is reshaped by evolution over time. Through compensatory mutations, both the plasmid and the host chromosome can acquire mutations that resolve the initial fitness cost. This represents a trajectory across this vast landscape, where one plasmid-host pair can migrate from a low-fitness valley to areas where the plasmid either becomes natural or beneficial, effectively locking in the resistance plasmid (29, 30).
3. The environment axis: the overall shape of the landscape is modulated by external environment factors. Physiochemical and nutrient availability, as well as chemical factors like sub-MIC concentrations of antibiotics, can significantly affect the fitness cost of any plasmid. It has also been shown that external factors also affect the rates of horizontal gene transfer (63, 64, 75).

Therefore, the success of any plasmid cannot be predicted by analyzing any single dimension in isolation. It is the intersection of these three axes that determines the success rate of a plasmid and ultimately the fate of any resistance gene. Thus, the persistence of a plasmid is a function of its ability to find a suitable host (host axis), in a permissive environment (environment axis), and where the pathways of compensatory mutations can be efficiently engaged (time axis).

### A call for a new, predictive framework

This new paradigm calls for a shift in perspective in how we approach the study and surveillance of AMR. The traditional way of measuring fitness cost in a laboratory strain under controlled conditions is a poor predictor of its real-world epidemiological success (10). To move to a truly predictive science, we need a system-level approach where it integrates all three dimensions of the landscape.

The future lies in developing multi-scale computational models that can forecast a plasmid’s potential success. Such models would need to integrate diverse data streams. These models need to account for genomic data to predict any genetic conflict between the host and the plasmid. It also needs to consider metabolic data to uncover patterns in the host physiological state to understand how the host becomes adapted to plasmid burdens. Lastly, a model of this scale also needs to take environmental data parameters, such as temperature, nutrient availability, and the chemical state of the environment.

By leveraging advances in machine learning and systems biology, it may become possible to build models that, given a novel plasmid and a specific host genome, can predict the likely fitness outcome under a range of environmental conditions. This would represent a crucial step forward, moving the field from a reactive posture—tracking resistance after it has already spread—to a proactive one.

## OUTSTANDING QUESTIONS AND FUTURE DIRECTIONS

The establishment of the plasmid-host fitness landscape as a central paradigm resolves many long-standing questions about the persistence of mobile resistance but also brings new, nuanced questions into sharp focus. This paradigm moves the field beyond simply cataloging resistance genes and toward a more dynamic, predictive science. To accelerate this transition, future research should be prioritized to address the following outstanding questions that emerge from a landscape perspective.

1. Can we develop "anti-evolution" drugs that target compensatory pathways?
  This review has detailed how bacteria rely on specific and often predictable evolutionary pathways to ameliorate the cost of plasmid carriage (19, 20, 25). This raises the tantalizing possibility of developing drugs that do not kill bacteria, but instead block these compensatory mutations. Such a molecule would act as a “plasmid destabilizer,” locking in the initial fitness cost and ensuring that the ARG-carrying plasmid is rapidly outcompeted and lost from the population once antibiotic pressure is removed.
2. Can we use machine learning to predict plasmid-host compatibility from genomic data alone?
  Now that we understand fitness is an emergent property of a specific plasmid-host pairing, the next frontier is prediction. A major research effort should be focused on developing machine learning models that can predict the fitness outcome of a novel plasmid-host encounter. By training algorithms on large data sets of plasmid and host genome sequences, coupled with experimental fitness measurements, it may be possible to identify the specific genetic motifs and interaction networks that signal a high-risk “compatible” pair (10).
3. How do fitness landscapes operate in complex, multi-species microbial communities?
  The majority of foundational studies on plasmid fitness have been conducted in well-defined, single-species laboratory systems (19, 20, 30). However, in nature, plasmids exist within complex, multi-species communities like the gut microbiome or soil. A critical unknown is how the principles of the fitness landscape operate in these environments. For instance, how does the presence of a “refuge host” population alter the selective pressure on other species (12)?
